# Factors associated with gestational weight gain: a cross-sectional survey

**DOI:** 10.1186/s12884-018-2112-7

**Published:** 2018-12-03

**Authors:** Edyta Suliga, Wojciech Rokita, Olga Adamczyk-Gruszka, Grażyna Pazera, Elżbieta Cieśla, Stanisław Głuszek

**Affiliations:** 1 Department of Nutrition and Dietetics, Faculty of Medicine and Health Sciences, Jan Kochanowski University, Kielce, Poland; 2 Department of Gynecological and Obstetric Prophylaxis, Faculty of Medicine and Health Sciences, Jan Kochanowski University, Kielce, Poland; 3 Clinic of Neonatology at the Regional Polyclinic Hospital, Kielce, Poland; 4 Department of Developmental Age Research, Faculty of Medicine and Health Sciences, Jan Kochanowski University, Kielce, Poland; 5 Department of Surgery and Surgical Nursing with the Scientific Research Laboratory, Faculty of Medicine and Health Sciences, Jan Kochanowski University, Kielce, Poland

**Keywords:** Dietary patterns, Body mass index, Excessive weight gain

## Abstract

**Background:**

The aim of this study was to describe the dietary patterns in pregnant women and determine the association between diet factors, pre-pregnancy body mass index, socio-demographic characteristics and gestational weight gain.

**Methods:**

The analysis was conducted on a group of 458 women. Cut-off values of gestational weight gain adequacy were based on recommendations published by the US Institute of Medicine and were body mass index-specific. Logistic regression analysis was used to assess the risk of the occurrence of inadequate or excessive gestational weight gain. Dietary patterns were identified by factor analysis.

**Results:**

Three dietary patterns characteristic of pregnant women in Poland were identified: ‘unhealthy’, ‘varied’ and ‘prudent’. The factor associated with increased risk of inadequate gestational weight gain was being underweight pre-pregnancy (OR = 2.61; *p* = 0.018). The factor associated with increased risk of excessive weight gain were being overweight or obese pre-pregnancy (OR = 7.00; *p* = 0.031) and quitting smoking (OR = 7.32; *p* = 0.019). The risk of excessive weight gain was decreased by being underweight pre-pregnancy (OR = 0.20; *p* = 0.041), being in the third or subsequent pregnancy compared to being in the first (OR = 0.37; *p* = 0.018), and having a high adherence to a prudent dietary pattern (OR = 0.47; *p* = 0.033).

**Conclusions:**

Women who were overweight or obese pre-pregnancy and those who quit smoking at the beginning of pregnancy should be provided with dietary guidance to prevent excessive gestational weight gain.

**Electronic supplementary material:**

The online version of this article (10.1186/s12884-018-2112-7) contains supplementary material, which is available to authorized users.

## Background

Abnormal gestational weight gain (GWG) is currently a serious obstetric problem. The prevalence of inadequate GWG varies among populations. In the US, GWG was *within the recommended range for 32% of women giving birth to full-term babies*. In 48% of cases, the increase in weight was higher, and in 21% of the cases, weight gain was lower than that recommended by the US Institute of Medicine (IOM) [1, 2]. A survey conducted between 2006 and 2015 on over 18,000 women in rural Pennsylvania showed that only 25.3% of women in this population gained weight within the recommended range – 21.3% gained an amount below and 52.9% gained an amount above the range in the IOM guidelines [3]. In a group of over 14,000 Italian women, the recommended GWG was found in 40.8%, in 30.1% of the women, GWG was lower, and in 29.1%, it was higher than the guidelines [4]. In German studies, 37.0% of women had excessive and 27.4% had inadequate GWG, according to the US IOM criteria [5]. Studies conducted in Poland showed that 40 to 48% of patients attain a GWG above the IOM guidelines and 14 to 23% attain a lower GWG [6, 7].

Abnormal GWG can have significant importance for both short-term pregnancy outcomes [1, 8, 9] and for the long-term health of the offspring [10–13] and the mother [8, 14, 15]. Health risks related to inadequate weight gain during pregnancy involve, first and foremost, a greater risk of premature birth and a low birth weight baby and/or intrauterine hypotrophy and, consequently, an increased risk of mortality and morbidity [1, 9, 16]. Excessive weight gain is indicated as a risk factor for giving birth to a high birth weight baby compared with its gestational age [9, 17], giving birth to a baby with macrosomia [4, 9, 18], gestational diabetes [19, 20], pregnancy-induced hypertension [21, 22], caesarean delivery [23, 24], longer infant hospital stays [22] and the persistence of a higher postpartum weight for the mother after childbirth, which predisposes one to obesity later in life [8, 14, 15, 25, 26].

Maternal pre-pregnancy body mass index (BMI) [1, 27], diet [28–30], physical activity [27, 29], smoking status [31] and socio-demographic factors [1, 32–34] are listed as the main determinants of GWG. Women who were overweight or obese prior to pregnancy were significantly more likely to exceed weight guidelines [1, 27, 34]. Tielemans et al. [35] confirmed that specific dietary patterns (DP) may play a role in early pregnancy but are not consistently associated with GWG. In the meta-analysis carried out by Streuling et al. [28], five studies suggested significant positive associations between energy intake and GWG, whereas three found no significant associations. Women who remain physically active during their pregnancies have a lower risk for excessive weight gain [27, 29]. However, some of the studies did not confirm any significant associations between physical activity and GWG [36, 37]. Current smokers are at an increased risk for insufficient weight gain, and former smokers are at an increased risk for excess GWG, compared to women who have never smoked [31]. It is worth noting that some studies did not confirm the relationship between smoking and GWG [38]. Therefore, the relationship between lifestyle and GWG is inconclusive.

Studies of the impact of socio-economic factors on GWG show that in the US, women with lower incomes gained more than the recommended weight compared to women with higher incomes [31, 39]. Women with less than a high school education had higher odds of inadequate GWG [40]. Huynh et al. showed that having a college or higher education was associated with a decreased GWG for non-Hispanic white women, but an increased GWG for Hispanic women [41]. Abbasalizad Farhangi states that in Iran women with high educational attainment have a significantly higher GWG compared with low-educated women [32]. As we can see, the presented research results are ambiguous and indicate that the risk of incorrect GWG in different populations may be determined by various cultural and socio-economic factors. Thus, they should always be taken into account when examining weight gain in pregnant women.

Very few papers have been published on GWG within the Polish female population [6, 7, 42], and the relationship between dietary patterns and GWG has not been studied thus far. Dietary patterns are specific to particular populations, since they may vary with age, sex, ethnicity, cultural traditions, socioeconomic status, and food availability. Research confirms that there are important differences in dietary habits between and within Eastern and Western European countries [43, 44]. Thus, it is important to analyse them in different populations. Therefore, we have formulated a hypothesis that specific dietary patterns can be identified The aim of this study was to describe the dietary patterns in pregnant Polish women and determine the association between dietary factors, pre-pregnancy body mass index, smoking, socio-demographic characteristics and gestational weight gain.

## Materials and methods

### Study setting and population

This cross-sectional study was conducted within 12 months between 2014 and 2015. The data were collected through a self-administered questionnaire and completed with information from the women’s medical documentation collected by a trained midwife.

The material consisted of the data of 505 women who, after childbirth, were patients of the Clinic of Obstetrics and Gynaecology of the Provincial Polyclinic Hospital in Kielce, Poland. The women who participated in the study aged 18–42 had given birth to a healthy child (without birth defects) and had labour that occurred after a full-term pregnancy, i.e., after the 37th week of pregnancy. The following patients were excluded from further analysis: four women with twin pregnancies, 18 patients whose labour occurred before the completion of the 37th week, and 25 patients lacking data. Finally, the analysis was conducted on a group of 458 women. This group comprised 12.1% of all women delivering during the year at the hospital where the study was conducted.

### Study measures

The diet of the study participants during their most recent pregnancy was evaluated with the use of the authors’ original semi-quantitative questionnaire of food frequency (FFQ) (Additional file 1). It was based on the principles of adequate nutrition of pregnant women, developed at the Institute of Food and Nutrition in Warsaw [45]. It has been used in previous studies, the results of which have been published in several papers [7, 46, 47]. The questionnaire was completed by all the women at the same time, i.e., 1 day before their scheduled discharge from the hospital. The FFQ assessed the consumption of vegetables (in total), fruit (in total), legumes, meat and meat-based products, sea fish, milk and dairy products, total grain food, whole grains, total fat, sweets and cakes, fast food, total drinks, fruit juice, sugary fizzy drinks, coffee, beer and/or wine, and strong liquors. The questions regarding the intake of particular groups of products and drinks during pregnancy concerned the number of standard portions consumed in a day and a week. The size of a portion was determined according to the guidelines defined in the literature [45]. The questionnaire also contained questions related to some eating habits, i.e., the number of meals consumed in a day, snacking between meals, and sweetening with sugar.

The nutritional status of the subjects before pregnancy was assessed on the basis of self-reported data on height and weight before pregnancy, which were used to calculate the BMI. The following groups were distinguished: the underweight group (BMI < 18.5 kg/m^2^; *N* = 37), those with a normal body mass (18.5–24.9 kg/m^2^; *N* = 377), and those who were overweight or obese (BMI ≥ 25.0 kg/m^2^; *N* = 44). Due to the small number of obese participants (*N* = 6), the analyses were carried out in the combined groups of overweight and obese. The data concerning prenatal body mass and duration of the pregnancy were obtained based on the analysis of medical documentation. The total GWG of each woman was calculated as the difference between their last weight prior to delivery minus their weight before pregnancy. A self-administered questionnaire was used to collect information about age, parity (first, second, third, or subsequent labour), the occurrence of persistent vomiting during the pregnancy (no; yes, in the 1st trimester of pregnancy; or yes, during the whole pregnancy), smoking (never, passive, current smoker or quit smoking after conception), the place of residence (large city: ≥50 thousand residents; small city: < 50 thousand residents; or countryside), education (lower than secondary school, secondary school, or university), and always having adequate money to buy necessary food (yes or no).

### Data analysis

Factor analysis by principal component analysis was used to determine dietary patterns (DP). Information about the frequency of consumption of certain portions of products included in the FFQ was the basis for the selection of variables for the analysis. The data obtained in this way were transformed into daily food intake and then normalized using the z-score procedure. The Bartlett Test of Sphericity and the Kaiser–Meyer–Olkin Measure of Sampling Adequacy were used to assess data adequacy for factor analysis. The applied procedure excluded sweetening with sugar from the analysis, which did not show any relationship with any other items. Factors were rotated using the Varimax procedure to improve the interpretation of the results. The number of the determined factors was established using Kaiser criterion (> 1). Additionally, it was verified by Cattell’s criterion (scree plot test). Food items were retained in the pattern if the factor loading value was above 0.30. On this basis, three factors were determined, and their own values were calculated for each of them. In the dietary pattern analysis, the majority of respondents are in the ‘middle’- range of the indicator values ​​and have little characteristic nutritional features, which causes difficulty in interpretation. Therefore we divided these dietary pattern data into quartiles. Study participants were assigned to the factor for which they obtained the highest score (i.e., 4th, the highest quartile).

The total GWG of each woman was calculated as the difference between their last weight prior to delivery minus their weight before pregnancy. GWG was classified as inadequate, adequate, or excessive. Cut-off values of gestational weight gain adequacy were based on the recommendations published by the US Institute of Medicine and were BMI-specific [1]. Adequate weight gain in women who were underweight before pregnancy should be 12.5–18.0 kg; in women with normal body weight it should be 11.5–16.0 kg; and in women who are overweight and obese it should be 7–11.5 kg and 5–9 kg, respectively. In the three GWG categories, for categorical data (place of residence, level of education, adequate money to buy food, persistent vomiting, smoking, and dietary patterns I-III), the structure indicators were calculated. The chi-square test was calculated for each factor to evaluate the relationships between structure indicators. Distributions of normality were checked for continuous variables (age and BMI of the subjects). The significance of differences between the means in three GWG categories was assessed by means of one factor analysis of variance (ANOVA) or Kruskal-Wallis one-way analysis of variance, depending on the distribution of the characteristics and the homogeneity of the variance. Inter-group differences between the means were evaluated by means of a post-hoc Bonferroni test. To assess the risk of the occurrence of inadequate or excessive GWG, logistic regression analysis was used (OR and 95% CI). Two models, crude and adjusted, were calculated for both categories of weight gain (inadequate and excessive). Adequate GWG was adopted as the reference level (1.0). The covariates included in the models were age, place of residence, education, having adequate money to buy necessary food, pre-pregnancy BMI, persistent vomiting, smoking, and dietary patterns. The following reference levels were adopted: for place of residence it was large city, for education it was university, for having adequate money to buy necessary food it was the answer “yes”, for pre-pregnancy BMI it was normal (18.5–24.9 kg/m^2^), for age it was < 30 years, for persistent vomiting it was the answer “no”, for smoking it was never smokers, and for DP it was the lowest quartile (Q1).

Because all of the patients in the group with low GWG marked the same answer category for the variable “having adequate money to buy necessary food”, the OR indicator and 95% CI were not calculated. The statistical analysis was carried out using SPSS software version 16.0. The *p* values *p* < 0.05 were considered statistically significant.

## Results

Table 1 presents the characteristics of the socio-demographic variables and lifestyle of the study participants, depending on the GWG category. The women with excessive GWG were characterized by higher pre-pregnancy BMI compared with women with adequate and inadequate weight gain. Moreover, it was found that excessive GWG was significantly more often related to giving up smoking in the first weeks of pregnancy. Women who continued.

**Table 1:** The characteristics of the study participants in three categories of GWG (N%; X ± SD)

Variables	Gestational weight gain			*p* value
	Inadequate (*N* = 100) 9.49 ± 2.01 kg	Adequate (*N* = 207) 13.76 ± 1.66 kg	Excessive (*N* = 151) 19.60 ± 4.04 kg	
Age (X ± SD)	29.19 ± 4.87	29.62 ± 4.93	30.43 ± 3.80	0.090^A^
Pre-pregnancy BMI (X ± SD)	20.92 ± 2.46	21.32 ± 2.50	22.82 ± 3.57^a,b^	**< 0.001** ^A^
Place of residence				
village	33 (33.0)	80 (38.6)	61 (40.4)	0.700^C^
town	19 (19.0)	42 (20.3)	31 (20.5)	
city	48 (48.0)	85 (41.1)	59 (39.1)	
Education				
lower than secondary school	6 (6.0)	10 (4.8)	12 (7.9)	0.729^C^
secondary school	30 (30.0)	55 (26.6)	39 (25.8)	
university	64 (64.0)	142 (68.6)	100 (66.2)	
Having adequate money to buy necessary food			1	
yes	100 (100.0)	202 (97.6)	47 (98.0)	0.306^C^
no	0 (0.0)	5 (2.4)	3 (2.0)	
Smoking				
non-smokers	55 (55.0)	120 (58.0)	86 (57.0)	**0.003** ^C^
passive smokers	28 (28.0)	65 (31.4)	42 (27.8)	
current smokers	15 (15.0)	20 (9.7)	10 (6.6)	
women who quit smoking after conception	2 (2.0)	2 (1.0)	13 (8.6)	
Persistent vomiting				
no	60 (60.0)	119 (57.5)	98 (65.3)	0.411^C^
yes, in the 1st trimester of pregnancy	36 (36.0)	75 (36.2)	48 (32.0)	
yes, during the whole pregnancy	4 (4.0)	13 (6.3)	4 (2.7)	
Parity				
first	54 (54.0)	102 (49.3)	80 (51.5)	0.243^C^
second	36 (36.0)	69 (33.3)	56 (37.1)	
third or subsequent labour	10 (10.0)	36 (17.4)	15 (9.9)	
Unhealthy DP				
Q1	21 (21.0)	52 (25.1)	42 (27.8)	0.715^C^
Q2	27 (27.0)	57 (27.5)	32 (21.2)	
Q3	28 (28.0)	46 (22.2)	39 (25.8)	
Q4	24 (24.0)	52 (25.1)	38 (25.2)	
Varied DP				
Q1	26 (26.0)	59 (28.5)	30 (19.9)	0.246^C^
Q2	30 (30.0)	50 (24.2)	35 (23.2)	
Q3	25 (25.0)	49 (23.7)	39 (25.8)	
Q4	19 (19.0)	49 (23.7)	47 (31.1)	
Prudent DP				
Q1	20 (20.0)	47 (22.7)	48 (31.8)	**0.036** ^C^
Q2	30 (30.0)	47 (22.7)	39 (25.8)	
Q3	18 (18.0)	57 (27.5)	37 (24.5)	
Q4	32 (32.0)	56 (27.1)	27 (17.9)	

Q represents quartile; DP represents dietary pattern; ^A^ represents one-way ANOVA; and ^C^ represents the chi-square test. The numbers in **bold** indicate statistically significant results; ^a^ represents the Bonferroni post hoc test *p* < 0.001 (adequate–excessive; and ^b^ represents the Bonferroni post hoc test p < 0.001 (inadequate–excessive)

to smoke during pregnancy more often had an inadequate GWG, compared with those who quit smoking. In women with excessive GWG, a lower adherence to prudent patterns was noted in comparison to other participants in the study. Other lifestyle elements and socio-demographic characteristics did not differ in each GWG category.

Three DPs were identified, which, in total, accounted for 33.2% of the variance (Table 2). These patterns describe mutual associations between consumed food groups, and they were named according to the food groups loading highest on the respective DP. The first one, ‘unhealthy’, is characterized by high intakes of fast food, alcohol, sugary fizzy drinks, cake, sweets, and coffee. The second pattern, ‘varied’, is characterized by high intakes of fruit and vegetables, fruit juice, fats, grain products, milk and dairy products, meat and meat-based products, and snacking between meals. The third pattern, ‘prudent’, was characterized by high consumption of whole grains, vegetables, legumes, sea fish, milk and dairy products and by having meals more often, as well as drinking greater total amounts of liquids; it was negatively correlated with snacking between meals.

**Table 2:** Factor-loading matrix for major dietary patterns*

Food groups	Factor I Unhealthy	Factor II Varied	Factor III Prudent
Fast food	0.682		
Beer or wine	0.682		
Strong liquors	0.600		
Sugary fizzy drinks	0.652		
Sweets, cakes	0.487		
Coffee	0.410		
Fruit		0.661	
Fat in total		0.612	
Cereals in total		0.556	
Snacking between meals		0.468	−0.412
Vegetables		0.443	0.421
Milk and dairy products		0.363	0.355
Fruit juice		0.330	
Meat and meat-based products		0.300	
Whole grains			0.710
Number of meals a day			0.502
Legumes			0.497
Sea fish			0.382
Drinks in total			0.305
Percentage of variance explained (%)	13.4	11.1	8.7

*Values <0.30 were excluded for simplicity

In the crude model, the risk of inadequate GWG was significantly higher in women underweight before pregnancy (OR = 2.14; *p* = 0.037) (Table 3). An increased risk of excessive GWG was positively associated with a pre-pregnancy BMI equal to ≥25 kg/m^2^ (OR = 6.44; *p* < 0.001), with giving up smoking (OR = 9.07; *p* = 0.004), and with a high score (Q4) of varied DP (OR = 1.89; *p* = 0.036). A lower risk of excessive GWG was associated with being underweight pre-pregnancy compared with having normal body mass (OR = 0.17; *p* = 0.020) and a high adherence (Q4) to prudent DP (OR = 0.047; *p* = 0.016).

**Table 3:** Factors determining the risk of inadequate and excessive GWG (unadjusted)

Variables	Gestational weight gain		
	Inadequate (*N* = 100)	Adequate (*N* = 207)	Excessive (*N* = 151)
Age			
< 30 years	1.0		1.0
≥30 years	1.02 (0.63–1.64)	1.0	1.36 (0.89–2.08)
*p* value	0.940		0.159
Pre-pregnancy BMI			
< 18.5 kg/m^2^	**2.14** (1.05–4.36)	1.0	**0.17** (0.04–0.76)
*p* value	**0.037**		**0.020**
18.5–24.9 kg/m^2^	1.0		1.0
≥25.0 kg/m^2^	0.85 (0.22–3.28)	1.0	**6.44** (2.87–14.42)
*p* value	0.812		**<0.001**
Place of residence			
city	1.0		1.0
town	0.80 (0.42–1.53)	1.0	1.06 (0.60–1.88)
*p* value	0.502		0.833
village	0.73 (0.43–1.25)	1.0	1.10 (0.69–1.76)
*p* value	0.253		0.695
Education			
university			
secondary school	1.21 (0.71–2.06)	1.0	1.01 (0.62–1.63)
*p* value	0.484		0.978
lower	1.33 (0.46–3.82)	1.0	1.70 (0.71–4.10)
*p* value	0.595		0.234
Having adequate money to buy necessary food			
yes	1.0		1.0
no	-*	1.0	0.82 (0.19–3.51)
*p* value	–		0.794
Smoking			
non-smokers	1.0		1.0
passive smokers	0.94 (0.54–1.62)	1.0	0.90 (0.56–1.45)
*p* value	0.824		0.670
current smokers	1.64 (0.78–3.44)	1.0	0.70 (0.31–1.57)
*p* value	0.193		0.383
women who quit smoking after conception	2.18 (0.30–15.89)	1.0	**9.07** (1.20–41.23)
*p* value	0.441		**0.004**
Persistent vomiting			
no	1.0		1.0
yes, in the 1st trimester of pregnancy	0.95 (0.58–1.58)	1.0	0.78 (0.50–1.22)
*p* value	0.848		0.272
yes, during the whole pregnancy	0.61 (0.19–1.95)	1.0	0.37 (0.12–1.18)
*p* value	0.405		0.094
Parity			
first	1.0		1.0
second	0.99 (0.59–1.66)	1.0	1.03 (0.65–1.64)
*p* value	0.956		0.884
third or subsequent labour	0.49 (0.20–1.20)	1.0	0.61 (0.30–1.27)
*p* value	0.118		0.186
Unhealthy DP			
Q1	1.0		1.0
Q_2_	1.17 (0.59–2.32)	1.0	0.69 (0.38–1.26)
*p* value	0.647		0.230
Q_3_	1.51 (0.75–3.01)	1.0	1.05 (0.58–1.89)
*p* value	0.245		0.872
Q_4_	1.14 (0.57–2.30)	1.0	0.91 (0.51–1.62)
*p* value	0.709		0.737
Varied DP			
Q1	1.0		1.0
Q_2_	1.36 (0.71–2.60)	1.0	1.38 (0.74–2.55)
*p* value	0.349		0.309
Q_3_	1.16 (0.59–2.26)	1.0	1.57 (0.85–2.88)
*p* value	0.667		0.149
Q_4_	0.88 (0.44–1.78)	1.0	**1.89** (1.04–3.42)
*p* value	0.721		**0.036**
Prudent DP			
Q1	1.0		1.0
Q_2_	1.50 (0.75–3.01)	1.0	0.81 (0.45–1.46)
*p* value	0.253		0.486
Q_3_	0.74 (0.35–1.56)	1.0	0.64 (0.36–1.13)
*p* value	0.432		0.124
Q_4_	1.34 (0.68–2.65)	1.0	**0.47** (0.26–0.87)
*p* value	0.396		**0.016**

The numbers in **bold** indicate statistically significant results; DP represents dietary pattern; Q represents quartile; * represents the number of individuals in the category ‘having adequate money to buy necessary food, answer no’, and the reference group is “adequate” GWG

In the adjusted model, the factor increasing the risk of inadequate GWG was being underweight pre-pregnancy (OR = 2.61; *p* = 0.018), whereas this risk was significantly lower in the third or subsequent pregnancy compared with the first one (OR = 0.39; *p* = 0.042) (Table 4). The factors increasing the risk of excessive GWG were being overweight or obese pre-pregnancy (OR = 7.00; *p* = 0.031) and giving up smoking in the first weeks of pregnancy (OR = 7.32; *p* = 0.019) (Table 4); however, the risk of excessive GWG was decreased by being underweight pre-pregnancy (OR = 0.20; *p* = 0.041), being the third or subsequent pregnancy compared to the first (OR = 0.37; *p* = 0.018) and with having high adherence (Q4) to the prudent DP (OR = 0.47; *p* = 0.033).

**Table 4:** Factors determining the risk of inadequate and excessive GWG (adjusted)

Variables	Gestational weight gain		
	Inadequate (*N* = 100)	Adequate (*N* = 207)	Excessive (*N* = 151)
Age			
< 30 years	1.0		1.0
≥30 years	1.30 (0.70–2.44)	1.0	1.67 (0.97–2.88)
*p* value	0.410		0.065
Pre-pregnancy BMI			
< 18.5 kg/m^2^	**2.61** (1.17–5.78)	1.0	**0.20** (0.04–0.94)
*p* value	**0.018**		**0.041**
18.5–24.9 kg/m^2^	1.0		1.0
≥25.0 kg/m^2^	0.88 (0.21–3.73)	1.0	**7.00** (2.87–17.08)
*p* value	0.872		**<0.001**
Place of residence			
city	1.0		1.0
town	0.70 (0.34–1.44)	1.0	1.27 (0.65–2.49)
*p* value	0.330		0.488
village	0.68 (0.36–1.29)	1.0	1.13 (0.62–2.08)
*p* value	0.240		0.689
Education			
university	1.0		1.0
secondary school	1.41 (0.75–2.65)	1.0	0.86 (0.46–1.59)
*p* value	0.289		0.627
lower	1.82 (0.52–6.42)	1.0	1.70 (0.54–5.39)
*p* value	0.350		0.365
Having adequate money to buy necessary food			
yes	1.0		1.0
no	-*	1.0	1.09 (0.20–6.04)
*p* value	–		0.924
Smoking			
non-smokers	1.0		1.0
passive smokers	0.88 (0.47–1.63)	1.0	0.96 (0.54–1.70)
*p* value	0.683		0.879
current smokers	1.64 (0.64–4.20)	1.0	1.08 (0.39–2.98)
*p* value	0.305		0.878
women who quit smoking after conception	2.08 (0.24–18.02)	1.0	**7.32** (1.39–38.56)
*p* value	0.506		**0.019**
Persistent vomiting			
no	1.0		1.0
yes, in the 1st trimester of pregnancy	0.90 (0.51–1.60)	1.0	0.63 (0.37–1.08)
*p* value	0.732		0.094
yes, during the whole pregnancy	0.48 (0.13–1.79)	1.0	0.44 (0.12–1.60)
*p* value	0.276		0.214
Parity			
first	1.0		1.0
second	0.91 (0.47–1.77)	1.0	0.74 (0.42–1.29)
*p* value	0.789		0.289
third or subsequent labour	**0.39** (0.16–0.96)	1.0	**0.37** (0.16–0.84)
*p* value	**0.042**		**0.018**
Unhealthy DP			
Q1	1.0		1.0
Q_2_	1.43 (0.68–3.00)	1.0	0.62 (0.26–1.04)
*p* value	0.342		0.066
Q_3_	1.45 (0.65–3.21)	1.0	0.97 (0.49–1.95)
*p* value	0.362		0.942
Q_4_	1.15 (0.49–2.71)	1.0	1.00 (0.48–2.08)
*p* value	0.754		0.996
Varied DP			
Q1	1.0		1.0
Q_2_	1.53 (0.76–3.09)	1.0	1.41 (0.70–2.85)
*p* value	0.234		0.332
Q_3_	1.04 (0.49–2.21)	1.0	1.38 (0.69–2.78)
*p* value	0.916		0.367
Q_4_	0.83 (0.36–1.80)	1.0	1.85 (0.92–2.78)
*p* value	0.640		0.085
Prudent DP			
Q1	1.0		1.0
Q_2_	1.27 (0.60–2.69)	1.0	0.70 (0.35–1.39)
*p* value	0.532		0.301
Q_3_	0.62 (0.28–1.39)	1.0	0.72 (0.38–1.39)
*p* value	0.247		0.329
Q_4_	1.38 (0.66–2.91)	1.0	**0.47** (0.23–0.97)
*p* value	0.394		**0.033**

The numbers in **bold** indicate statistically significant results, DP represents dietary pattern, Q represents quartile, * represents the number of individuals in the category ‘having adequate money to buy necessary food, answer “no”, and the reference group is “adequate” GWG

## Discussion

To the best of our knowledge, this is the first paper to identify the DP of pregnant women in Poland. In women in the highest quartile of the ‘prudent’ pattern, characterized by high intakes of whole grains, vegetables, legumes, sea fish, milk and dairy products, and avoiding snacking between meals, the risk of excessive GWG was significantly lower. Dietary patterns, as is well known, can differ between countries and populations, which accounts for the fact that the results obtained are not always comparable with the results of studies by other authors. Lai et al. found that the highest tertile of plant-based protein food intake was associated with a 60% lower likelihood of inadequate GWG and a 34% lower likelihood of excessive GWG [48]. Stuebe et al. [49] showed that a vegetarian diet in the first trimester is inversely associated with excessive GWG. Studies conducted in Norway showed that adherence to a regional diet rich in fruits and vegetables, potatoes, whole grains, fish, game, milk, and drinking water during pregnancy may facilitate the maintenance of optimal GWG in normal-weight women [50]. Tielemans et al. found that specific DP can play a significant role in early pregnancy but is not subsequently related to GWG [35]. However, these authors noted more moderate GWG in normal-weight women with higher scores on the ‘nuts, high-fibre cereals, and soy’ pattern than in women with a low score for this pattern. Wrottesley et al. noted that an increased intake of a traditional diet pattern high in whole grains, vegetables, legumes, traditional meats, and of a decreased intake of sugar and fat was related to a lower risk of excessive GWG [51]. Chuang et al. confirmed that appropriate GWG was related to intentional planning of meals and snacks [52]. Additionally, the results of intervention studies quite explicitly show that rationale eating habits among pregnant women contributes to the optimization of their GWG [29, 36, 53].

Several authors have shown that an unhealthy DP is significantly correlated with excessive GWG [49, 51, 54], although our study did not confirm such a relationship. Unambiguous scores related to the abovementioned associations may have occurred because dietary patterns vary according to age, ethnicity, culture, and other lifestyle factors. In a study conducted among Swedish women, their intakes of caloric beverages, snacks, fish, and bread were positively related to excessive GWG [30]. Uusatilo et al. found that greater adherence to a ‘fast food’ DP, characterized by high intakes of fast food items such as hamburgers and pizza, as well as sweets, soft drinks and added sugar, was positively associated with GWG [54].

The results of our study showed that the factors related to inadequate GWG were pre-pregnancy BMI, smoking and parity. Being underweight pre-pregnancy was significantly correlated with gaining too little weight compared to gaining weight within the guidelines. It is commonly emphasized that being underweight results in a higher risk of insufficient GWG, whereas being overweight and/or obese at the time of conception is related to a higher risk of excessive gain [1, 6, 27, 30]. Fontaine et al. showed that 33 to 50% of healthy weight women and 50 to 75% of overweight and obese women had excessive GWG [55]. Heerman et al. found excessive GWG in 55.0% of mothers who were overweight before pregnancy, in 43.7% of those who were obese, and only in 37.5% of mothers who were underweight before pregnancy [56]. The results of our analyses agree with those of other authors.

The factor that strongly determined GWG in our study was quitting smoking. The results of several other papers also confirmed that women who quit smoking after conception gain much more weight than women who had never smoked [57–59]. Research carried out in the general population suggests, that heavy smokers (≥25 cigarettes per day) and those who were obese before quitting gain the most weight [60]. Although quitting smoking is a significant health-promoting change in lifestyle, it can also lead to negative metabolic consequences. Bush et al., on the basis of a literature review, found that weight gain related to quitting smoking results mainly from a decline in resting-state basal metabolism [61]. Furthermore, nicotine in cigarettes is an appetite suppressant. Depriving oneself of it results in an increase in appetite and in emotional eating, calorie misperception, and a greater craving for sweets, which may lead to excessive calorie intake.

The results of our study are consistent with reports of other authors that primiparous women gain more weight and that the probability of the occurrence of excessive GWG is higher compared to their multiparous counterparts [36, 62]. Only Hill et al. did not find any differences in GWG between primiparous and multiparous women [63]. In several papers, there were no significant associations reported between age and GWG [64–66], which is in agreement with the results of our study. In other studies, it is emphasized that a lower GWG is present in older women [1, 36, 67]. However, the results obtained by these authors usually concern women aged up to > 35 and not ≥30, as is the case in our analysis.

Despite the large number of publications regarding the association between socioeconomic status and GWG, the literature is inconsistent [32, 33, 39–41, 68, 69]. Women with low income have an increased risk for both excessive and inadequate GWG [69]. The reason for this is largely due to a lack of understanding of the importance of a healthy diet during pregnancy as well as limited access to healthy food. However, Guilloty et al. found that socio-demographic characteristics were not associated with GWG, which is consistent with the results of our studies [70]. Among the participants in our study, there were few women (only 1.75%) who reported a lack of adequate money to buy necessary food. Therefore, the results obtained should be considered with great caution. In most studies, a higher risk of gaining weight outside of the recommendations was found in women with a lower level of education [32, 40, 71, 72]. Cohen et al. suggested that higher education plays a role in healthier GWG for some, but not all, groups of pregnant women [33]. In most cases, higher education was associated with a lower chance of inadequate GWG. Educational attainment was also associated with excessive GWG; however, ethnicity and pre-pregnancy overweight status both modified this association, sometimes in different directions. In addition, the correlation analysis among the participants in our study suggests that a high adherence to the unhealthy DP negatively correlated with a higher level of education (Spearman’s rank correlation coefficient = − 0.28; *p* < 0.05; data not shown). Thus, although there is no direct relationship between education and GWG, this factor may affect the GWG indirectly by modifying the diet. This issue requires confirmation with a larger group of women.

The main limitation of the study is the lack of data concerning the physical activity of the women subjects, which can have an influence on GWG [27, 29]. However, some of the studies did not confirm any significant associations between physical activity and GWG [36, 37]. Moreover, for the evaluation of food intake, a non-validated, authors’ original semi-quantitative questionnaire was applied. Standard questionnaires are very long and extensive, and in the opinion of the management of the hospital, they could be an excessive burden for women shortly after delivery. They could also contribute to lower response rates among women. The results obtained should also be treated with caution because of the small number of obese participants (*N* = 6; 1.31%), as well as the small number of those who were overweight (*N* = 38; 8.30%). However, it is known that in the Polish population, excessive body mass occurs least often among young women. In a representative group (i.e., 1129 female university students aged 20–24 years from the south of Poland), it was noted that 6.5% were overweight and only 0.5% were obese (BMI was calculated on the basis of the measurements of body height and mass) [73]. There was also a small number of women who quit smoking during pregnancy (*N* = 17; 3.7%). Therefore, the smoking results should also be approached with great caution.

## Conclusions

Three dietary patterns, characteristic of pregnant women in Poland, were identified: ‘unhealthy’, ‘varied’ and ‘prudent’. The risk of excessive GWG was lower in women with a high adherence to the prudent DP, which was characterized by a high intake of vegetables, legumes, whole grains, sea fish, milk, and dairy products, as well as a larger amount of total drinks, the consumption of a larger number of planned meals, and avoiding snacking between meals. The factors that was positively associated with a higher risk of excessive GWG in the study population were quitting smoking at the beginning of pregnancy and excessive body mass at the time of conception. The risk of inappropriate GWG (both too low and excessive) was smaller in the third and subsequent pregnancies compared to the first. Being underweight pre-pregnancy increased the risk of inadequate GWG and decreased the risk of excessive weight gain. Women who were overweight or obese pre-pregnancy and those who quit smoking at the beginning of pregnancy should be provided dietary guidance to prevent them from excessive GWG. Prevention programmes for pregnant women, developed to optimize their GWG, should include socio-economic factors, cultural traditions, and dietary patterns that are specific to a given population.

## Additional file

Additional file 1: The Food Frequency Questionnaire (FFQ). The authors’ original semi-quantitative questionnaire of food frequency consumption during the most recent pregnancy. (DOCX 21 kb)

## Abbreviations

BMI: Body mass index
DP: Dietary pattern
FFQ: Food frequency questionnaire
GWG: Gestational weight gain
IOM: Institute of medicine
OR: Odds Ratio
Q: Quartile
US: United States of America
