# Supplementary material for: Factors associated with gestational weight gain: a cross-sectional survey
Source: BMC Pregnancy Childbirth. 2018 Dec 3;18:465. doi: 10.1186/s12884-018-2112-7 (PMC6276162; doi:10.1186/s12884-018-2112-7)
Supplement: Supplementary file 1 — The Food Frequency Questionnaire (FFQ). The authors’ original semi-quantitative questionnaire of food frequency consumption during the most recent pregnancy. (DOCX 21 kb) [file 12884_2018_2112_MOESM1_ESM.docx]

Additional File 1.

THE FOOD FREQUENCY QUESTIONNAIRE (FFQ)

Dear Madame,

This questionnaire aimed to assess your diet during this most recent pregnancy.

Please put a cross next to the selected answer ǀ_x.

1. How many planned meals did you eat a day during this most recent pregnancy?

ǀ_ 1-2 meals ǀ_ 3 meals ǀ_ 4 meals ǀ_ 5 meals ǀ_ 6 or more meals

2. Did you snack between planned meals?

ǀ_ No ǀ_ Less frequently than once a day ǀ_ Once a day ǀ_ A few times a day

3. Did you suffer from persistent vomiting during this pregnancy?

ǀ_ No ǀ_ Yes, in trimester one ǀ_ Yes, during the whole pregnancy.

4. How many portions of fruit did you eat in a day? (1 portion equals 100 g, e.g., 1 small apple or half a big one, half an orange, 1 mandarin, 1 kiwi, 7 strawberries, 5 plums, etc.)

ǀ_⁬ I did not eat any fruit

ǀ_⁬ I ate fruit less frequently than once a day

ǀ_⁬ I ate 1 – 2 portions a day

ǀ_⁬ 3 portions a day

ǀ_⁬ 4 portions a day

ǀ_⁬ 5 or more portions a day

5. How many portions of vegetables did you eat in a day? (1 portion equals 100 g of vegetables, e.g., 1 medium-sized tomato, 2 medium-sized carrots, 1 medium-sized boiled potato)

ǀ_⁬ I did not eat any vegetables

ǀ_⁬ I ate vegetables less frequently than once a day

ǀ_⁬ I ate 1 – 2 portions a day

ǀ_⁬ 3 portions a day

ǀ_⁬ 4 portions a day

ǀ_⁬ 5 portions a day

ǀ_⁬ 6 or more portions a day

6. How many portions of legumes did you eat during your pregnancy? (1 portion equals about 50 g of dry beans, peas, soy, lentils)

ǀ_⁬ I did not eat eating any legumes

ǀ_⁬ I ate them less frequently than once a week

ǀ_⁬ I ate 1 - 3 portions a week

ǀ_⁬ I ate 4 - 6 portions a week

ǀ_⁬ 1 portion a day

ǀ_⁬ 2 or more portions a day

7. How many portions of milk or dairy products did you eat a day? (1 portion equals e.g., 1 glass of milk, yoghurt, kefir, 100 g of white cheese, 2 slices of hard cheese)

ǀ_⁬ I did not eat any dairy products

ǀ_⁬ I ate dairy products less frequently than once a day

ǀ_⁬ I ate 1 – 2 portions a day

ǀ_⁬ 3 portions a day

ǀ_⁬ 4 portions a day

ǀ_⁬ 5 or more portions a day

8. How many portions of meat products did you eat every day? (1 portion is equal to e.g., 150 g of fish or meat, 2 eggs, part of a portion can consist of cold cured meat)

ǀ_⁬ I did not eat any meat products

ǀ_⁬ I ate meat products less frequently than once a day

ǀ_⁬ I ate 1 – 2 portions a day

ǀ_⁬ 3 portions a day

ǀ_⁬ 4 portions a day

ǀ_⁬ 5 or more portions a day

9. How often did you eat sea fish (e.g., mackerel, herring, sardines, salmon, etc.)?

ǀ_⁬ I did not eat any sea fish

ǀ_⁬ I ate sea fish less frequently than once a week

ǀ_⁬ I ate 1 portion a week

ǀ_⁬ 2-3 portions a week

ǀ_⁬ 4-6 portions a week

ǀ_⁬ Every day I have been eating at least one portion

10. How many portions of grains did you eat altogether every day? (1 portion is equal to e.g., 1 slice of bread 50 g, 1 bread roll, 4 pieces of crispbread, 3 spoons of groats, rice or pasta – after boiling or 2 spoons of cereal).

ǀ_⁬ I did not eat any grains

ǀ_⁬ I ate grains less frequently than once a day

ǀ_⁬ I ate 1 portion a day

ǀ_⁬ 2-3 portions a day

ǀ_⁬ 4-5 portions a day

ǀ_⁬ 6-7 portions a day

ǀ_⁬ 8 or more portions a day

11. How many of these portions were comprised of whole grains e.g., dark (wholemeal) bread, crispbread, coarse-cut groats (buckwheat, pearl barley, dark rice)

ǀ_⁬ I did not eat any whole grains

ǀ_⁬ I ate whole grains less frequently than once a day

ǀ_⁬ I ate 1 portion a day

ǀ_⁬ 2-3 portions a day

ǀ_⁬ 4-5 portions a day

ǀ_⁬ 6-7 portions a day

ǀ_⁬ 8 or more portions a day

12. How often did you eat cakes or sweets? (1 portion equals about 50 g of biscuits, a bar, half a chocolate, a small portion of cake)

ǀ_⁬ I did not eat any cakes or sweets

ǀ_⁬ I ate 1-3 portions a week

ǀ_⁬ I ate 4-6 portions a week

ǀ_⁬ I ate 1 portion a day

ǀ_⁬ 2-3 portions a day

ǀ_⁬ 4-5 portions a day

ǀ_⁬ 6 or more portions a day

13. How many portions of fat did you eat a day? (1 portion equals about 1 big spoon or 3 teaspoons of butter, margarine, oil, lard – for frying or raw)

ǀ_⁬ I did not eat any fat

ǀ_⁬ I ate no more than half a portion a day

ǀ_⁬ I ate 1 portion a day

ǀ_⁬ 2 portions a day

ǀ_⁬ 3 portions a day

ǀ_⁬ 4 or more portions a day

14. In this most recent pregnancy, how often did you eat: hamburgers, hot-dogs, pizza, chips, kebabs or other “fast food”?

ǀ_ None at all

ǀ_ I ate “fast food” less frequently than once a month

ǀ_⁬I ate “fast food” less frequently than once a week

ǀ_⁬I ate 1-3 portions a week

ǀ_⁬I ate 4-6 portions a week

ǀ_ Every day

15. How many glasses of liquids altogether did you drink a day?

ǀ_ fewer than 4 glasses ǀ_ 4-5 glasses ǀ_ 6-7 glasses ǀ_ 8 or more glasses

16. How many glasses of fruit juice (100% juice, not fruit beverages) or vegetable juice did you drink during pregnancy?

ǀ_⁬ I did not drink any fruit or vegetable juice

ǀ_⁬ I drank less than one glass a week

ǀ_⁬ 1-3 glasses a week

ǀ_⁬ 4-6 glasses a week

ǀ_⁬ 1 glass a day

ǀ_⁬ 2 or more glasses a day

17. Did you sweeten with sugar your drinks (e.g., tea) or consumed dishes?

ǀ_ Yes ǀ_ No

18. How many glasses of sugary fizzy drinks did you drink when pregnant?

ǀ_⁬ I did not drink any sugary fizzy drinks

ǀ_⁬ I drank less than one glass a week

ǀ_⁬ 1-3 glasses a week

ǀ_⁬ 4-6 glasses a week

ǀ_⁬ 1 glass a day

ǀ_⁬ 2 or more glasses a day

19. Have you tried to lose weight during this most recent pregnancy?

ǀ_ Yes ǀ_ No

20. How many cups of coffee (including instant coffee) did you drink when pregnant?

ǀ_⁬ I did not drink any coffee

ǀ_⁬ I drank less than 1 cup a week

ǀ_⁬ 1-3 cups a week

ǀ_⁬ 4-6 cups a week

ǀ_⁬ 1 cup a day

ǀ_⁬ 2 or more cups a day

21. How many glasses of beer or glasses of wine did you drink during pregnancy?

ǀ_⁬ I did not drink any beer or wine at all

ǀ_⁬ I drank occasionally, less than one glass a month

ǀ_⁬ I drank 1 - 3 beer glasses / wine glasses a month

ǀ_⁬ 1 - 3 beer glasses / wine glasses a week

ǀ_⁬ 4 - 6 glasses a week

ǀ_ 1 or more glasses a day

22. How many glasses of strong liquor (cognac, vodka, etc.) did you drink when pregnant?

ǀ_⁬ I did not drink any strong liquor

ǀ_⁬ I drank occasionally, less than one glass a month

ǀ_⁬ I drank 1 - 3 glasses of strong liquor a month

ǀ_⁬ 1 - 3 glasses of strong liquor a week

ǀ_⁬ 4 - 6 glasses a week

ǀ_ 1 or more glasses a day

Thank you for completing the questionnaire!
